# Phosphor-Necrosis

**Published:** 1860-01

**Authors:** 


### ARTICLE XII.

### *Phosphor-Necrosis.*

Dr. Markoe presented two specimens removed from the same patient, illustrating phosphor-necrosis of the jaw, a disease which was now attracting a great deal of attention, and the pathological anatomy of which differed in a considerable number of points from that of necrosis, occurring under ordinary circumstances. While under other circumstances necrosis produces suppuration on the outside, the separation of the dead bone and the formation of an involucrum, here a totally different circumstance occurs. Instead of the separation of a sequestrum, we find the surrounding parts secreting bone immediately around the dead portion. The dead bone becomes incrusted with pumice-stone-like material, which adheres very firmly to the bone, so much so that it can hardly be separated from it. These two specimens illustrate very beautifully this variety of disease. They were taken from a man who labored in a match factory. He had a tooth extracted, and at that point the mischief commenced. At the end of five months he was subjected to an operation, and a portion of the jaw removed. The wound healed up, and shortly afterward he went back to the factory. The disease then attacked the other side of the jaw, and this was removed, and also a portion of the upper jaw.

The unfortunate feature of these cases is, that this pumice-stone exudation represents a reparative attempt, or rather it represents what would be accomplished, if the dead bone were taken away. In that case the periosteum would secrete new bone, which would replace the old bone. But when this pumice-stone exudation takes place, there is no reproduction of the bone. In this case, it is now eighteen months since the operation, and there is no formation of new bone tissue. The upper jaw appeared to be simply necrosed. There was none of this exudation, or at least so little, that it was not noticed.

The patient is doing well, and although both halves of the lower jaw and one side of the upper jaw have been removed, his face is very shapely.

Dr. Sayer inquired whether the rule was a general one, that in these cases of removal of the jaw for phosphor-necrosis, no attempts at new bony formation took place afterward. He thought quite the contrary was the case, and remarked that Dr. Wood, who had perhaps more experience on this subject than any other man in this country, in one case removed both upper jaws, when the bones grew in again, and the patient had placed upon them a set of artificial teeth. He would defy any one to detect that the jaws had been removed in that patient, so complete was the deception. He hoped that Dr. Wood would favor the society with some remarks on this subject.

Dr. Wood did not desire the society to go into a discussion of this matter now. He would, however, on some future evening, bring some beautiful specimens, showing perfect reparation in cases where the bone had been destroyed by phosphorus. He would suggest that Drs. Markoe and Parker also bring their specimens, and then the society could go into a general discussion of the subject.

[*Report Proc's N Y. Path'l Soc. in Med. & Surg. Rep.*

